# Heat Shock Protein 70 Genes Are Involved in the Thermal Tolerance of *Hippodamia variegata*

**DOI:** 10.3390/insects15090678

**Published:** 2024-09-08

**Authors:** Qing Yang, Yanhui Lu

**Affiliations:** 1 State Key Laboratory for Biology of Plant Diseases and Insect Pests, Institute of Plant Protection, Chinese Academy of Agricultural Sciences, Beijing 100193, China; yangqingzzr@126.com; 2 Doctoral Work Laboratory, Department of Agricultural and Animal Husbandry Engineering, Cangzhou Technical College, Cangzhou 061001, China; 3 Western Agricultural Research Center, Chinese Academy of Agricultural Sciences, Changji 831100, China

**Keywords:** *Hippodamia variegata*, transcriptome, heat shock protein, RNA interference

## Abstract

**Simple Summary:**

*Hippodamia variegata* is a key natural enemy of cotton-growing regions in northwestern China. In this study, transcriptome sequencing analysis was performed on *H. variegata*, after beetles were exposed to different temperatures (from 32 to 38 °C) for 24 h, using high-throughput sequencing technology. We found the largest number of differentially expressed genes (DEGs) in the 35 °C vs. 32 °C group (1151); the fewest DEGs were found in the 38 °C vs. 35 °C group (901), indicating that *H. variegata* expressed the largest number of newly mobilized genes under medium-high temperature (35 °C). Gene functional analysis showed that the DEGs were mainly involved in “Catalytic activity”, “Oxidoreductase activity”, “Metabolic pathways”, and “Longevity regulating pathway-multiple species” gene groups. The results from qRT-PCR were consistent with the transcriptome data, confirming their reliability. Finally, the RNAi results showed that adult survival, longevity, and fecundity were lower in the group in which gene expression of the heat shock proteins (*Hsp70-01* and *Hsp68*) was suppressed than in the control group (injection ds-*GFP*) at all the experimental temperatures (32, 35, and 38 °C). Our results indicate the important role of the heat shock proteins (*Hsp70-01* and *Hsp68*) in resistance to high-temperature stress in *H. variegata* and provide a molecular basis for analyzing the thermotolerance mechanism of *H. variegata*.

**Abstract:**

Previous studies have shown that the survival and reproduction of *Hippodamia variegata* are increasingly harmed by progressive increases in temperature (from 32 °C to 35 °C and 38 °C). In this study, transcriptome sequencing analysis was performed on *H. variegata*, after being exposed to different temperatures (from 32 to 38 °C) for 24 h, using high-throughput sequencing technology. We found the largest number of differentially expressed genes (DEGs) in the 35 °C vs. 32 °C group (1151) followed by the 38 °C vs. 32 °C group (1054) and then the 38 °C vs. 35 °C group (901), indicating that *H. variegata* expressed the largest number of newly mobilized genes under medium-high temperature (35 °C). Gene functional analysis showed that a large number of DEGs were involved in “Catalytic activity”, “Oxidoreductase activity”, “Metabolic pathways”, and “Longevity regulating pathway-multiple species” gene groups. We randomly selected nine DEGs for validation using qRT-PCR. The results of qRT-PCR were consistent with the transcriptome data, confirming their reliability. Finally, the RNAi results showed that adult survival, longevity, and fecundity were lower in the group in which gene expression of the heat shock proteins (*Hsp70-01* and *Hsp68*) was suppressed than in the control group (injection ds-*GFP*) at all the experimental temperatures (32, 35, and 38 °C). Our results indicate the important role of the heat shock proteins (*Hsp70-01* and *Hsp68*) in resistance to high-temperature stress in *H. variegata* and provide a molecular basis for analyzing its thermotolerance mechanism.

## 1. Introduction

In recent years, the greenhouse effect has become increasingly severe. It is predicted that global temperatures could rise by up to 6 °C by the end of the 21st century [1]. As poikilotherms, insects have limited thermoregulation ability, and rising temperatures are a great challenge for them [2]. It is well known that high temperatures can affect all physiological and metabolic processes in insects [3], including survival, reproduction, feeding, and antioxidant capacity. Laboratory studies have shown that 31 °C was favorable for *Exochomus nigripennis*. Different temperatures (23, 27, 31, and 35 °C) affected the developmental time and mortality of *E. nigripennis* larvae, showing that the total larval development time was greatest at 23 °C and shortest at 35 °C. Mortality decreased significantly under temperatures from 23 to 31 °C, but increased at 35 °C [4]. Antioxidant enzymes remove excess oxygen free radicals [5,6]. However, high-temperature stress can inactivate antioxidant enzymes in insects, resulting in increased levels of active oxygen metabolites in insects and insect death [7,8,9]. Moreover, high temperatures also limit the geographical distribution of insects. Oliveira et al. [10] confirmed that temperature has a direct impact on the distribution of the ladybugs *Tenuisvalvae nota* and *Cryptolaemus monouzieri*. Also, studies have shown that *Cheilomenes sexmaculata* has gradually spread to higher latitudes in Japan with the increase in the annual mean temperature [11].

*Hippodamia variegata* (Coleoptera: Coccinellidae) is a significant natural enemy of aphids [12] that is widely distributed around the world [13,14]. Many studies have shown that elevated temperatures significantly affect the biological characteristics of ladybugs at various life stages, including developmental time, adult longevity, reproduction, predatory capacity, and distribution [10,15,16]. Kong et al. [17] showed that the ability of *H. variegata* adults to feed on cotton aphids was strongest at 35 °C, which was twice that measured at 17 °C. However, it seems that *H. variegata* beetles are more resistant to high temperatures and remain the dominant natural enemy population in Xinjiang farmland [18]. Our long-term field surveys have shown that *H. variegata* is widely distributed in the Xinjiang province, while another common ladybug, *Propylaea quatuordecimpunctata*, is only found in the northern-most, colder parts of the province. Our preliminary laboratory study found that the survival, longevity, fecundity, and antioxidant capacity of *H. variegata* were all higher than those of *P. quatuordecimpunctata* at 32, 35, and 38 °C [19].

Many studies have examined the effects of high temperatures on insect ecology, physiology, and biochemistry [9,20,21]. However, heat stress is a complex process [3,22,23] that can change the gene expression patterns of insects [7,24]. High-throughput sequencing can measure the expression levels of many genes and identify differentially expressed genes in the transcriptome [25], so it was widely used to study the response mechanism of insects to heat stress [26]. Shen et al. [27] found that, compared to commercial populations, natural populations of the bothriderid beetle *Dastarcus heliophoroides* better mobilize heat shock proteins (HSPs) and SOD and Pro enzymes under heat stress conditions. Similarly, Ma et al. [28] found that the HSP gene of *Apis mellifera* was upregulated under heat stress conditions. In addition, this technique has also been widely used in the study of the heat stress responses of other types of insects, such as silkworm *Bombyx mori* [29], *Tamarixia radiata* [30], and *P. quatuordecimpunctata* [31]. Recognizing which genes are regulated differently in insects under normal conditions and times of heat stress can help us identify the metabolic pathways involved and understand the underlying mechanisms by which insects adapt to heat stress.

In our study, high-throughput sequencing technology was used to reveal the mechanistic tolerance of *H. variegata* adults to different high temperatures (32, 35, and 38 °C). GO (Gene Ontology) and KEGG (Kyoto Encyclopedia of Genes and Genomes) enrichment analyses were used to understand the biological functions of differentially expressed genes (DEGs). Meanwhile, randomly selected genes were validated using quantitative reverse transcription polymerase chain Reaction (qRT-PCR) technology to verify the authenticity of transcriptome data. Finally, RNA interference (RNAi) technology was used to study the function of high-temperature-tolerant genes in *H. variegata*. These experimental data fill the gap in our understanding of the molecular biological mechanism of high-temperature tolerance in *H. variegata* and provide a theoretical basis for natural enemy conservation at high temperatures.

## 2. Materials and Methods

### 2.1. Insect Rearing

*H. variegata* and *Aphis gossypii* were collected from cotton fields (pesticide-free) at the Experimental Field Station of Shihezi University (44.32° N, 85.92° E) (Shihezi, China) in 2019. Key characteristics were used to determine the species of ladybug [32]. Next, the field-caught individuals were transferred to the Langfang Experimental Station of the Chinese Academy of Agricultural Sciences (CAAS; 39.53° N, 116.70° E) in Langfang, Hebei province. Ladybugs and cotton aphids were kept under laboratory conditions. Ladybugs were reared in plastainers (8 cm in diameter; 11.5 cm in height) within a controlled-climate chamber (RXZ500D, Ningbo Jiangnan Instrument Factory, Ningbo, China) and held at 32 ± 1 °C, 70 ± 5% RH, and a 16:8 h (L:D) photoperiod. Cotton aphids (*A. gossypii*) were reared on *Cucurbita pepo* (variety Xinzaoqing, Ji Nong Seed Co., Ltd., Tianjin, China) in screened cages (55 × 35 × 50 cm) in a greenhouse at 28–30 °C, 50 ± 5% RH, and a 16:8 h (L:D) photoperiod. An adequate number of cotton aphids (ca. 500 aphids per ladybug) were fed daily to ladybugs, and experiments were conducted after the ladybug population stabilized. *Cucurbita pepo* was replaced every five days to ensure the stabilization of the cotton aphid population.

### 2.2. High-Temperature Treatments

In earlier research, we tested actual summer temperatures typical of Xinjiang (32, 35, and 38 °C), and the results showed that *H. variegata* adults had different survival and reproduction rates under these different temperatures [19]. Therefore, we chose these temperatures as a realistic level for assessing the heat-resistant molecular mechanism of *H. variegata*.

One-day-old *H. variegata* adults (<12 h) were stored in a climate chamber (70 ± 5% RH and 16:8 h L:D) at 32 °C (control), 35 °C (medium-high temperature), or 38 °C (high temperature) for 24 h. Each temperature had three replicates, with two *H. variegata* adults in each replicate. So, six *H. variegata* adults were tested for each temperature. Finally, these *H. variegata* specimens were quickly frozen in liquid nitrogen for immediate RNA extraction.

### 2.3. RNA Extraction and Transcriptome Sequencing

The whole body of the heat-treated *H. variegata* adults was used for RNA extraction. Total RNA extraction was carried out using *TransZol* Up Plus RNA Kit (TransGen Biotech, Beijing, China), and the integrity, purity, and concentration of the RNA were verified with Agilent 2100 (Agilent Technologies, New York, NY, USA) and Nanodrop 2000 (IMPLEN, Munich, Germany) [33]. Subsequently, the checked RNA was used to build a library for sequencing. The specific steps were as follows: Magnetic beads with Oligo (dT) were used to enrich the mRNA. Then, the mRNA was broken up into short fragments by a fragmentation buffer. Single-stranded cDNA was synthesized by reverse transcription using random hexamers as triggers, and then double-stranded cDNA was synthesized by adding a buffer, dNTPs, and DNA polymerase I. Subsequently, we performed end repairs and added poly (A) and adapters on double-stranded cDNA purified with AMPure XP beads. We used AMPure XP beads to screen 250bp fragments of double-stranded cDNA and then performed PCR amplification to construct a cDNA library. Finally, the mRNA library was sequenced using Allwegene Technologies’ Illumina sequencing platform. The RNA-seq data are available in the NCBI/SRA database under BioProject accession number PRJNA1142879 (https://www.ncbi.nlm.nih.gov/sra/PRJNA1142879), accessed on 14 August 2024.

### 2.4. Quality Control, Analysis, and Functional Annotation

Raw data (raw reads) in fastq format were first processed through in-house perl scripts. The Trimmomatic software (v0.33) (SUNW Co., Ltd., CA, USA) was used to obtain clean data, removing reads with adapters, reads containing ploy-N (at a content higher than 10%), and low-quality reads (quality less than 20) from the raw data. Next, the Q20, Q30, GC content, and sequence duplication level of the clean data were calculated. All subsequent analyses were based on high-quality clean data. Trinity was used to assemble clean reads [34]. Finally, Tgicl [35] was used to cluster transcripts from each sample twice to remove redundancy. The final unigenes were then noted for subsequent analysis. The functions of all unigenes obtained above were annotated using seven major databases, such as Nr (NCBI non-redundant protein sequences), Nt (NCBI non-redundant nucleotide sequences), Pfam (Protein family), KOG/COG (Clusters of Orthologous Groups of proteins), Swiss-Prot (A manually annotated and reviewed protein sequence database), KO (KEGG Ortholog database), and GO (Gene Ontology).

### 2.5. Differentially Expressed Genes (DEGs) and Functional Enrichment Analysis 

The DESeq R software package (1.10.1) (AT&T Co., Ltd., Parlin, NJ, USA) was used for the differential expression analysis of the two groups [36]. DESeq provides statistical routines for determining differential expression in digital gene expression datasets using a model based on negative binomial distribution. The resulting *p* values were adjusted using Benjamini and Hochberg’s approach to control the false discovery rate. DESeq was used to label DEGs of adjusted *p* values < 0.05. Then, the DEGs were functionally analyzed by GO enrichment [37] and KEGG pathway enrichment [38].

### 2.6. Quantitative Real-Time PCR (qRT-PCR)

The qRT-PCR was used to verify the authenticity of transcriptome data. We randomly selected nine different genes (six P450 and three Hsp70) from the test results for qRT-PCR verification. *EF1ɑ* was selected as the internal reference gene [39]. The qRT-PCR primers designed by Primer3Plus required the measurement of amplification efficiency and specificity (Table 1). The first-strand cDNA synthesized by a reverse transcription kit (Tiangen Biotechnology Co., Ltd., Beijing, China) was mixed into a 20 μL reaction system by a fluorescence quantitative kit (Tiangen Biotechnology Co., Ltd., Beijing, China). Relative changes in gene expression were assessed using the 2^−ΔΔCt^ method [40]. Each treatment group was repeated three times, and each sample was technically repeated three times. All samples were used for sequencing.

The qRT-PCR reaction was performed on equally diluted template cDNA (five diluted gradients). We then drew a standard curve and calculated the amplification efficiency based on the Ct value of the qRT-PCR reaction results. The amplification efficiency should be between 90 and 105%, and the correlation coefficient (*R*^2^) of the standard curve should be greater than 98%.

### 2.7. Synthesis and Delivery of dsRNA

Based on the transcriptome recordings, we selected the DEGs of interest (*Hsp70-01* and *Hsp68*) as target genes for subsequent experiments. The RNA interference experiments first required the development of specific primers for the target gene (*Hsp70-01* and *Hsp68*) and the confirmation of the gene sequence used through PCR amplification and sequencing before their use. Next, the RNAi vector needed to be constructed. The 2× Taq PCR Master Mix for PAGE kit (Vazyme Biotech Co., Ltd., Nanjing, China) was used to amplify the target sequence with specific primers containing the T7 promoter sequence at the 5′ end (Table 1). The connecting product (PCR product + *pEASY*-T1 vector) was obtained using the *pEASY*-T1 Simple Cloning Kit (TransGen Biotechnology, Beijing, China) and then transformed into *E. coli* Trans1-T1 (TransGen Biotechnology, Beijing, China). Positive clones were sequenced for the subsequent synthesis of dsRNA. The *GFP* gene was used as a negative control (ds-GFP). Specific dsRNA sequences were produced using the T7 RiboMAX™ Express RNAi System (Promega, Madison, WI, USA), according to the manufacturer’s specifications. Next, the dsRNA was resuspended in RNase-free water. Then, 1% agarose-gel electrophoresis and a NanoDrop 2000 spectrophotometer (NanoDrop, Wilmington, DE, USA) were used for assessment and quantification. Finally, the dsRNA products were separated into tubes and stored at −80 °C until use.

One-day-old (<12 h) ladybug adults were used for the experiments. A PLI-100 p-injector (Harvard Apparatus, Holliston, MA, USA) was used to silence the expression of the target genes by the injection delivery of dsRNA. The ladybirds of the injected ds-*GFP* were used as controls. Within one hour of injection, living ladybugs were selected for subsequent experiments. For each target gene (*Hsp70-01* and *Hsp68*) and temperature (32, 35, and 38 °C), we collected two individuals injected as one independent sample at three different times (24, 48, and 72 h post injections) for a total of 18 samples. Each was analyzed using a technical triplicate by qRT-PCR. This experiment was then repeated three times. The experimental procedures used to separate the total RNA and qRT-PCR were the same.

### 2.8. Effects of RNA on Survival Rate and Fecundity

The survival and reproduction of adult ladybugs were observed at all experimental temperatures to determine the effects of the dsRNA treatment on the species. There were 3 replicates per treatment and 20 individuals per replicate. The adults were fed the cotton aphids daily (500 per cotton aphid). Observation continued until all adult ladybugs died.

### 2.9. Data Analysis

A one-way analysis of variance (ANOVA) was used to analyze the expression of candidate genes of *H. variegata* at different temperatures, and Tukey’s test was used to identify significant differences between specific temperatures in the expression of candidate genes in *H. variegata* (*p* < 0.05). Student *t*-test was used to analyze the differences between different treatment groups, and survival curves of different treatment groups were analyzed by the Kaplan–Meier log-rank test. All statistical analyses were conducted using the SPSS 25.0 (IBM Co., Ltd., Armonk, NY, USA) software and Microsoft Excel 2010 (Microsoft Co., Ltd., Redmond, WA, USA), while charts were generated using OriginPro 9.0 (OriginLab Co., Ltd., Northampton, MA, USA) and GraphPad Prism 8.0 (GraphPad Software Co., Ltd., La Jolla, CA, USA).

## 3. Results

### 3.1. Transcriptome Sequencing Quality Assessment and Functional Annotation

The transcriptome sequencing results are shown in Table 2. In all the tested samples, the error rate was 0.03%; the Q20 value was between 97.85% and 98.09%; the Q30 value was between 93.88% and 94.39%; and the GC content was between 40.61% and 41.44%. These results indicated that the sequencing library’s quality was good and that the sequencing data were accurate and reliable.

**Figure 1:**
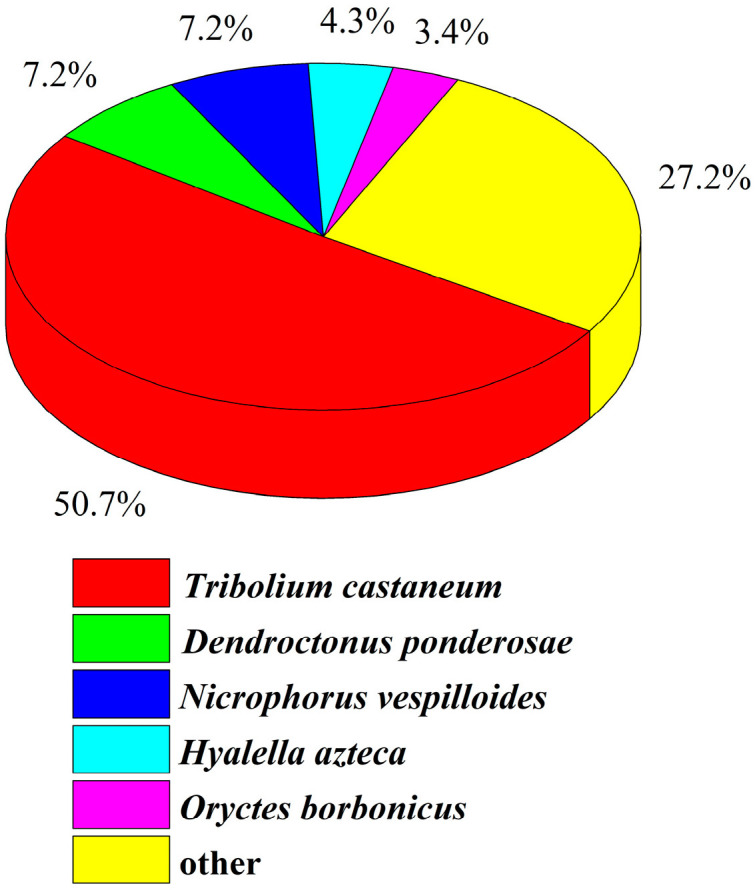
All unigene sequences for *Hippodamia variegata* that had blast annotations in the NR database were analyzed for species distribution.

After quality control and redundancy removal, 68,546 unigenes were detected in the samples of *H. variegata*. Seven databases were used to perform functional annotation on the unigene sequences obtained above. The largest number of unigenes was annotated by the NR database (23,616 unigenes), accounting for 34.4% of the total. A total of 762 unigene sequences were annotated by seven databases, accounting for 1.1% of the total (Table 3). The unigene sequences of *H. variegata* were compared and annotated with the NR database, which was used to understand the gene sequence similarity of *H. variegata* and other related species (Figure 1). We found that the genetic relationship between *H.variegata* and *Tribolium castaneum* is relatively close, with a similarity of up to 50.7% in their gene sequences.

### 3.2. Differentially Expressed Genes (DEGs)

**Figure 2:**
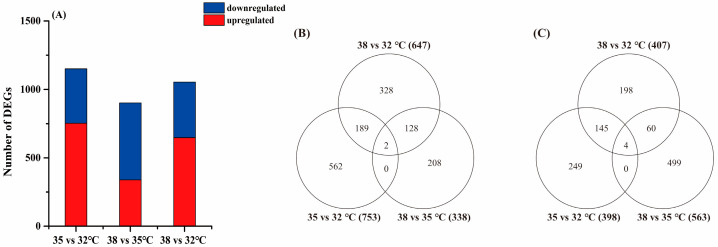
Differentially expressed genes (DEGs) in *Hippodamia variegata* under different degrees of temperature stress. (**A**) Total number of individual transcripts that were significantly up- or downregulated in different temperature groups. (**B**) Venn diagram illustrating the number of upregulated genes in the different temperature groups. (**C**) Venn diagram illustrating the number of downregulated genes in the different temperature groups.

In this study, the DEGs that were significantly different between the treatments were screened by their expression levels. The standard for DEG screening was a *q* value < 0.05 (after correction, FDR value). After statistical analysis, it was found that there was an overlapping relationship in the DEGs of *H. variegata* at three experimental temperatures (see Venn diagram in Figure 2). The largest number of DEGs (1151) was found in the 35 vs. 32 °C groups, including 753 upregulated genes and 398 downregulated genes. The minimum number of DEGs (901) was found in the 38 vs. 35 °C groups, including 338 upregulated genes and 563 downregulated genes. We speculate that *H. variegata* may have more genes mobilized at 35 °C and involved in defending against oxidative stress at 38 °C.

### 3.3. Identification of Genes Participating in Tolerance to High Temperatures 

**Figure 3:**
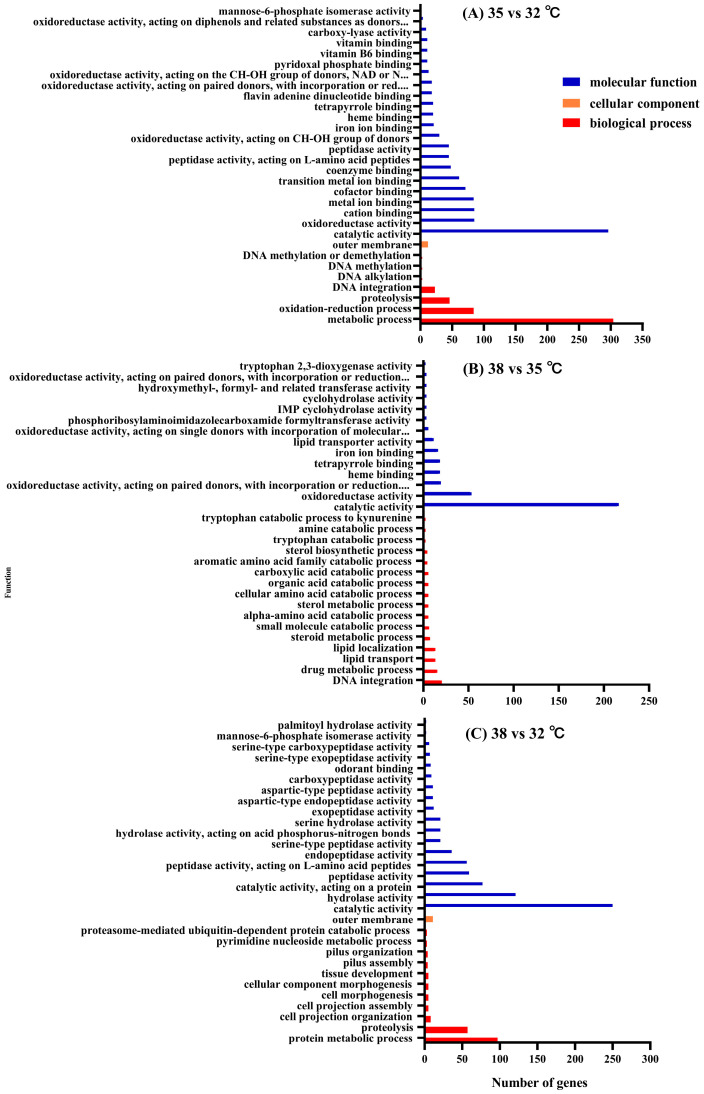
GO enrichment analysis of adult *Hippodamia variegata* under different levels of temperature stress.

A GO enrichment analysis can intuitively reflect the distribution of DEGs in the GO terms of biological processes, cellular components, and molecular functions. Therefore, a GO enrichment analysis was used to explore genes related to thermotolerance in *H. variegata*. This study shows the results of the top 30 GO terms’ enrichment significance (*p* value) of *H. variegata* (Figure 3). In all comparison groups, a large amount of DEGs were enriched in two categories: biological processes and molecular functions. In the 35 vs. 32 °C groups, a large amount of DEGs were enriched in the “transmembrane transport” pathway of biological processes and the “catalytic activity” and “oxidoreductase activity” pathways of molecular functions (Figure 3, Supplemental Table S1). Nevertheless, in the 38 vs. 35 °C groups, a large amount of DEGs were enriched in the “DNA integration” and “drug metabolic process” pathways of biological processes and the “catalytic activity” and “oxidoreductase activity” pathways of molecular functions (Figure 3, Supplemental Table S1). The above results indicate that, at 35 °C, the organism seems to focus on the transport of molecules and catalytic activities crucial for survival at high temperatures, while, at 38 °C, the response is oriented towards protection against oxidative damage and maintenance of the metabolism under stress conditions. The differences reveal possible reasons for differences in the response of *H. variegata* to different high temperatures (35 and 38 °C).

### 3.4. KEGG Enrichment Analysis of DEGs

**Figure 4:**
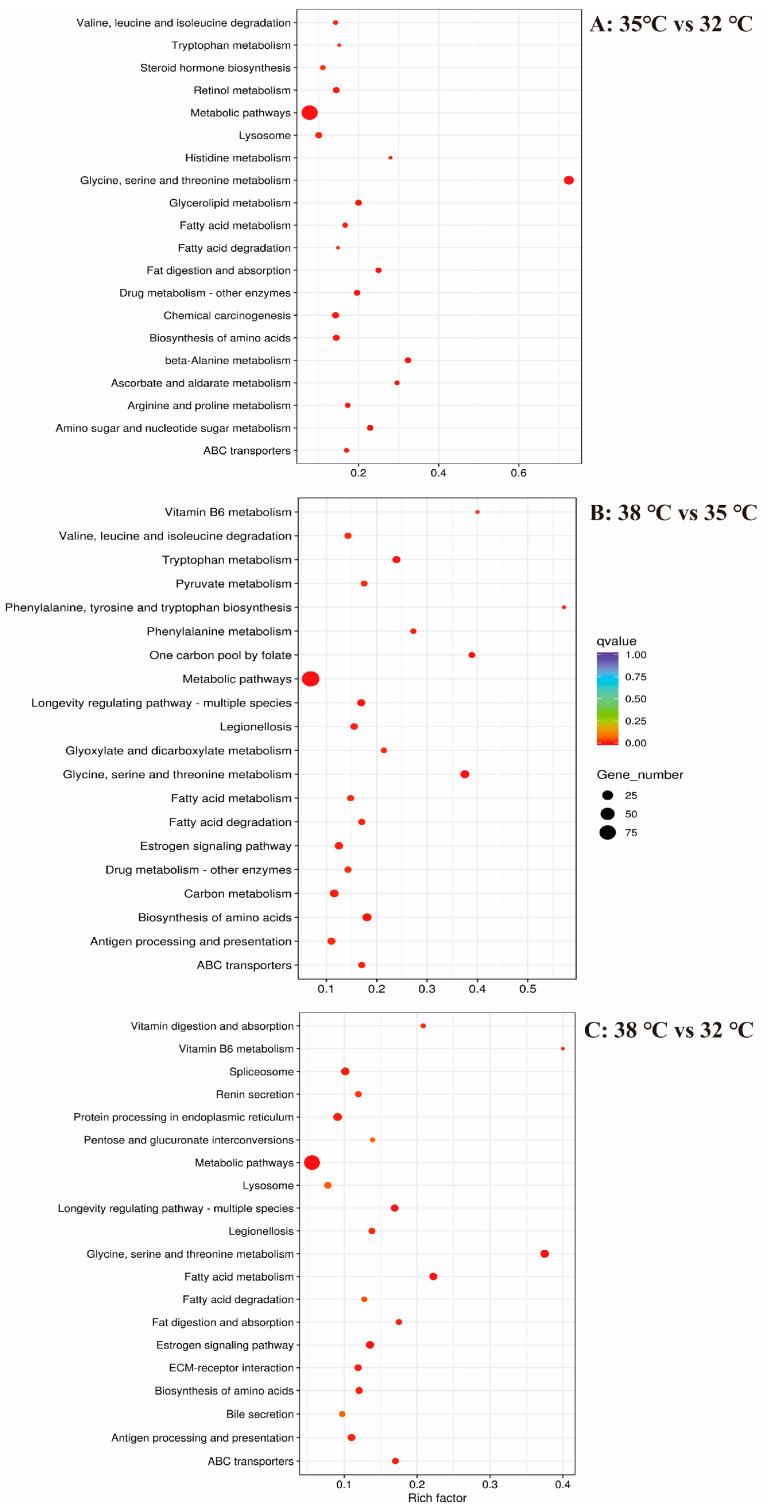
KEGG enrichment analysis of adult *Hippodamia variegata* under different levels of temperature stress.

The KEGG enrichment analysis was used to further explore the thermotolerance of *H. variegata*. For the KEGG metabolic pathways, the number of DEGs for the 35 vs. 32, 38 vs. 35, and 38 vs. 32 °C groups was increased to 216, 219, and 197, respectively. Here, we present the top 20 most significantly enriched pathways of *H. variegata* in the form of scatter plots (Figure 4), including “ABC transporters”, “Valine, leucine, and isoleucine degradation”, “Tryptophan metabolism”, “Fatty acid metabolism”, “Fatty acid degradation” “Metabolic pathways”, and “Drug metabolism–other enzymes”, which were co-enriched pathways in the 35 vs. 32 °C and 38 vs. 35 °C groups (Figure 4, Supplemental Table S2). An in-depth analysis was conducted on the DEGs involved in “Metabolic pathways”. Most of the DEGs (87 genes) were downregulated in the 35 vs. 32 °C groups, and only a few DEGs (15 genes) were upregulated in the 35 vs. 32 °C groups. Conversely, most of the DEGs (75 genes) were upregulated in the 38 vs. 35 °C groups, and only a few DEGs (15 genes) were downregulated in the 38 vs. 35 °C groups (Supplemental Table S2). 

### 3.5. Real-Time Fluorescence Quantitative PCR Validation

**Figure 5:**
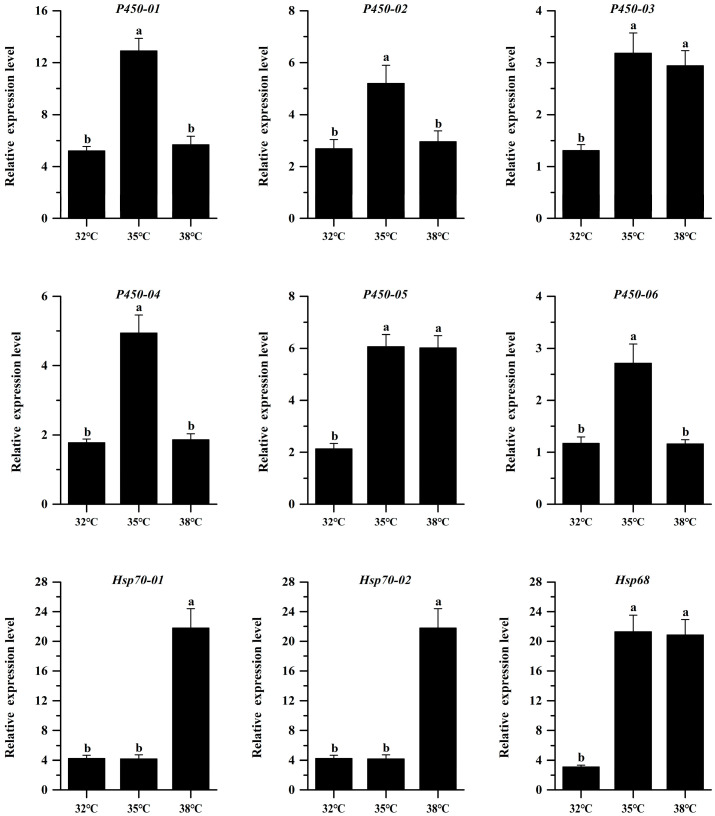
Real-time PCR validation of adult *Hippodamia variegata* DEGs under different levels of temperature stress. The results are shown as the means ± SE. Different letters above the bars indicate statistically significant differences between different temperatures in terms of gene expression (Tukey’s post hoc test; *p* < 0.05).

A large number of DEGs related to thermotolerance were found by functional enrichment analysis (Supplemental Tables S1 and S2). We randomly selected nine DEGs (six P450 and three Hsp70) for qRT-PCR verification. The FPKM values of the DEGs are shown in Supplemental Table S3. qRT-PCR was used to confirm the authenticity of transcriptome sequencing. First, we tested the primers, and the results showed that the amplification efficiency of all the primers was between 90% and 105% (Table 1). Moreover, the dissolution curves were all single peaks, demonstrating that these primers qualified as authentic. The qRT PCR results are consistent with the transcriptome sequencing (RNA Seq) (Supplemental Table S3, Figure 5), indicating that the transcriptome sequencing results were effective. High-temperature stress resulted in the upregulated expression of cytochrome P450 and heat shock protein Hsp70 in *H. variegata* (Tukey′s post hoc test: *p* < 0.001, Figure 5). Among them, the expression level of heat shock protein family genes continued to increase after high-temperature stress, so they were selected as the target genes for subsequent RNAi validation.

### 3.6. Hsp70 Genes Are Required for H. variegata Survival under High-Temperature Stress

The dsRNA of *Hsp70-01* and *Hsp68* was delivered by microinjection to the abdomen of *H. variegata* adults, which were then subjected to heat stress treatment at different experimental temperatures (32, 35, and 38 °C). The expression levels of heat shock protein genes (*Hsp70-01* and *Hsp68*) were measured by the qRT-PCR technique at 24, 48, and 72 h after microinjection, and the level of suppression was calculated. The results showed that the *Hsp70-01* and *Hsp68* genes of *H. variegata* were successfully suppressed within 72 h, with a significant reduction in their expression compared to the injection of ds-*GFP*, and the expression level was the lowest 48 h after injection (*p* < 0.001, Table 4). With an increase in temperature, the inhibition of gene expression increased. Under the highest temperature (38 °C), under which the *Hsp70-01* and *Hsp68* genes were suppressed for 48 h, the gene expression level decreased by 57.3% and 44.0%, respectively (*Hsp70-01*: *t* = 21.372, *df* = 4, *p* < 0.001; *Hsp68*: *t* = 31.938, *df* = 4, *p* < 0.001, Table 4).

**Figure 6:**
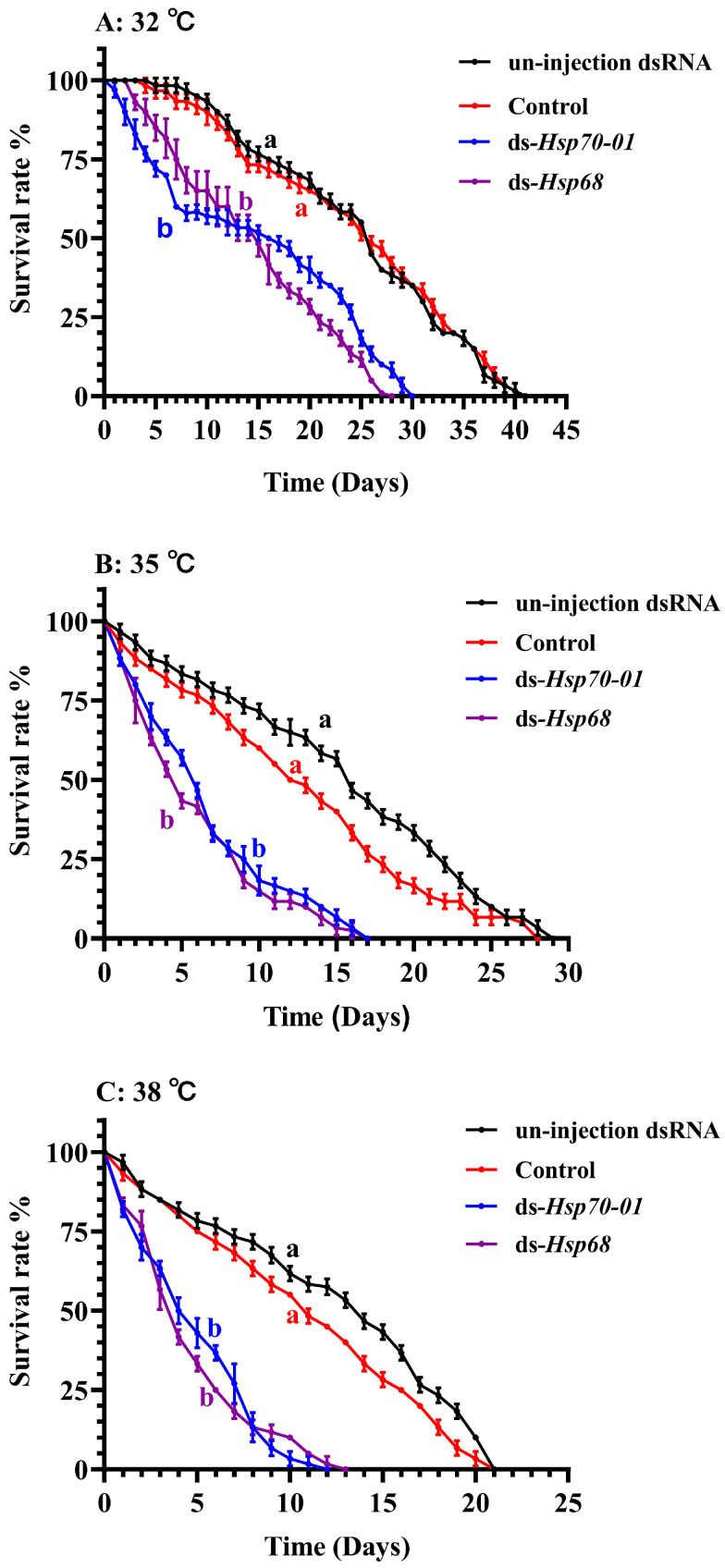
Adult survival rate of *Hippodamia variegata* under different temperatures when the *Hsp70-01* and *Hsp68* genes were either fully functional (the control) or partly suppressed by the injection of dsRNA-RNAi gene sequences. Different letters with the same color indicate that the corresponding color curve is different from the control (log-rank, *p* < 0.05, individual = 20).

We confirmed that the use of dsRNA-mediated RNAi technology could successfully reduce the expression of the *Hsp70-01* and *Hsp68* genes in *H. variegata* adults, allowing us to create treatments with and without normal levels of expression of these genes. We then measured adult beetle survival at all three temperatures for beetles in normal and partly suppressed gene expression states. We found that the survival rate of *H. variegata* adults gradually decreased over time, but that this happened faster in groups in which the expression of the *Hsp70-01* and *Hsp68* genes had been partially suppressed compared to the control (injection ds-*GFP*) (*p* < 0.001, Figure 6). Also, the higher the temperature, the greater the rate of mortality from such suppression of the *Hsp70-01* and *Hsp68* genes (*p* < 0.001, Figure 6B,C).

**Figure 7:**
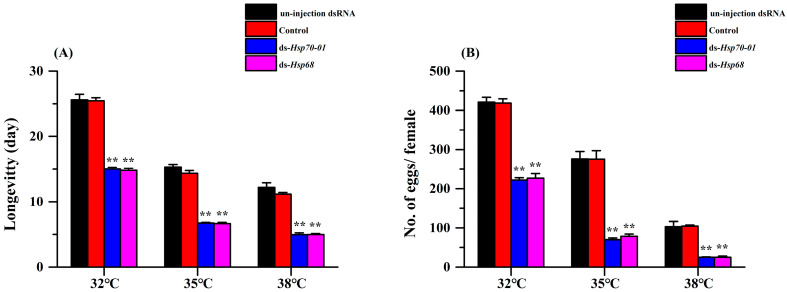
Average adult longevity (**A**) and fecundity (**B**) of *Hippodamia variegata* under different temperatures after the *Hsp70-01* and *Hsp68* genes were suppressed with dsRNA-RNAi gene sequences. The results are shown as the means ± SE. The control was injected with the ds-*GFP*. The asterisk (*) above each bar indicates significant differences (Student’s *t*-test, *p* < 0.05) between the dsRNA treatments and the control. **, *p* < 0.01.

As a consequence of higher rates of adult beetle mortality, when the expression levels of the *Hsp70-01* and *Hsp68* genes of *H. variegata* adults were suppressed with dsRNA-RNAi gene sequences, the average longevity at all experimental temperatures for beetles in the treated group was significantly shorter than for the control group (Figure 7A). Indeed, due to the suppression of these genes, longevity decreased by 41.3%, 56.0%, and 60.9% with the suppression of the *Hsp70-01* gene (32 °C: *t* = 20.558, *df* = 4, *p* < 0.001; 35 °C: *t* = 39.192, *df* = 4, *p* < 0.001; 38 °C: *t* = 17.010, *df* = 4, *p* < 0.001) and by 42.1%, 56.6%, and 59.2% with the suppression of the *Hsp68* gene (32 °C: *t* = 17.929, *df* = 4, *p* < 0.001; 35 °C: *t* = 35.859, *df* = 4, *p* < 0.001; 38 °C: *t* = 17.306, *df* = 4, *p* < 0.001), respectively, at the three experimental temperatures.

The fecundity of *H. variegata* adults was also reduced at all experimental temperatures (32 °C, 35 °C, and 38 °C) when the expression of the *Hsp70-01* and *Hsp68 genes* was suppressed (Figure 7B). Specifically, the oviposition rates of *H. variegata* adults decreased by 47.2%, 74.6%, and 76.0% with the suppression of the *Hsp70-01* gene (32 °C: *t* = 25.003, *df* = 4, *p* < 0.001; 35 °C: *t* = 18.287, *df* = 4, *p* < 0.001; 38 °C: *t* = 9.866, *df* = 4, *p* = 0.001) and by 46.1%, 71.6%, and 75.9% with the suppression of the Hsp68 gene (32 °C: *t* = 18.591, *df* = 4, *p* < 0.001; 35 °C: *t* = 15.485, *df* = 4, *p* < 0.001; 38 °C: *t* = 9.643, *df* = 4, *p* = 0.001), respectively, at the three experimental temperatures.

## 4. Discussion

Temperature is one of the most important factors affecting insect growth and development, feeding, physiology, and geographical distribution [41,42,43]. Previous studies have shown that *H. variegata* has different types of adaptability to different levels of high temperatures [19]. Therefore, we used transcriptome sequencing methods to explore the molecular mechanism supporting the responses of *H. variegata* to heat stress [44,45]. We found that about a third of the unigenes detected in the *H. variegata* samples were annotated in the Nr database (34.5%, 23,616 unigenes). Among these, the highest match (up to 50.7%) was with *T. castaneum*, which indicates that *H. variegata* has the highest homology with *T. castaneum*. Meanwhile, Lv et al. [46] showed that *T. castaneum* could complete generation development at 35 °C, which was similar to the high-temperature tolerance of *H. variegata*. We also found that the most DEGs (1151: 753 upregulated and 398 downregulated) were detected in the 35 vs. 32 °C groups, and there were fewer overlapping genes between the 35 and 38 °C groups. This indicates that the response mode of the genes regulated in the 35 vs. 32 °C groups was different from that of other groups. This result is similar to our previous transcriptome analysis of the high-temperature tolerance of *P. quatuordecimpunctata* [31]. The differences in gene expression between different temperature treatment groups indicate that the regulation mechanisms of *H. variegata* are different, which needs further study.

When insects are subject to heat stress, they evolve complex and diverse behavioral, physiological, biochemical, and molecular mechanisms to resist damage from unfavorable temperatures [9]. Among these defense mechanisms, heat shock proteins play a key role in preventing the deterioration of proteins and peptides in organisms [47]. The expression level of HSP genes increases significantly under high-temperature conditions [48,49,50]. Tang et al. [51] found that high temperatures (31, 33, and 35 °C) stimulate the increased expression of the *Fohsp60* and *Fohsp90* genes in *Frankliniella occidentalis* nymphs compared to 26 °C. In *Myzus persicae*, exposure to high temperatures (30, 35, and 40 °C) for 1 h significantly increased the expression level of the *MpHsp70a* gene compared to the 25 °C control [52]. Similarly, in our study, we also found that a high temperature (38 °C) significantly increased the expression of the Hsp68, *Hsp70-01*, and *Hsp70-02* genes in *H. variegata*. Heat shock proteins are chaperone molecules that can protect other proteins during heat stress, preventing thermal denaturation, the aggregation of substrate proteins, and abnormal protein degradation [53,54,55]. In terms of their functions, heat shock proteins include the constitutive heat shock protein (HSC) with stable expression levels and the induced heat shock protein (HSP), which can have high expression during periods of external stimulation [56,57]. Serine residues were found in the ATPase domain of heat shock proteins in the Hsp70 family of genes [58,59]. According to Yu et al. [60], this feature is unique to inducible Hsp70 (A), so we concluded that the heat shock protein found in this study belonged to the inducible Hsp70 (A) type. In addition, most oxidoreductases genes showed an upregulation trend after high-temperature stress. Previous studies also found that the activities of the antioxidative enzymes SOD and CAT in *H. variegata* increased under high-temperature stress [19], indicating that these genes play important roles in the high-temperature resistance process. Liu et al. [23] found that some antioxidation-related genes in crambid moth *Glyphodes pyloalis* (Walker) were significantly upregulated under heat stress (exposure to 40 °C for 4 h compared to 25 °C). Finally, the KEGG enrichment analysis found that most of the DEGs in “Metabolic pathways” were upregulated at 38 °C, suggesting that a high temperature (38 °C) may cause metabolic pathway disorders in insects [7].

Using population survival curves as our response variable and gene silencing as an intervention, we showed that, when the heat shock protein 70 family genes (*Hsp70-01* and *Hsp68*) of *H. variegata* were suppressed, the survival rate of a population of this ladybug declined significantly at all temperatures compared to the control, and this decreasing trend became more significant over time. This indicates that the heat shock protein 70 family genes (*Hsp70-01* and *Hsp68*) have protective effects against thermal stress in *H. variegata*. Similar phenomena have also been found in other studies. For example, when expression of the Hsp70 gene of the chrysomelid beetle *Agasicles hydrophila* was suppressed, the beetles’ survival rate under a high-temperature treatment (36 °C and 39 °C) was significantly lower than in control (injection ds-*GFP*) beetles, and this difference became more significant with increasing temperatures [61]. We also observed that the adult longevity and fecundity of *H. variegata* decreased significantly when expression of the heat shock protein 70 family of genes (*Hsp70-01* and *Hsp68*) was suppressed, at all experimental temperatures.

After pre-exposure at 32 °C and 35 °C, *Liriomyza sativae* showed a significant increase in the expression of the heat shock protein gene hsp70, increasing the leafminer’s thermotolerance, but the feeding and reproductive abilities of the resulting adults were significantly reduced [62]. When the MpHsp70a gene of *M. persicae* was suppressed, the sensitivity of the aphid to high temperatures (40 °C) increased significantly [52]. In a previous study, it was also found that high temperatures (35 and 38 °C) significantly reduced the survival and reproduction of *H. variegata* adults, although these temperatures also increased the expression level of the heat shock protein 70 family of genes (*Hsp70-01* and *Hsp68*) in adults. The trade-off between ladybug adaptation to high temperatures and heat shock protein gene induction remains to be further investigated [19].

We also confirmed that the heat shock proteins in the Hsp70 family (*Hsp70-01* and *Hsp68*) play an important role in high-temperature resistance in *H. variegata*. However, such resistance in insects is very complex and needs further exploration. At the same time, our previous results showed that, in addition to the Hsp70 family of genes (*Hsp70-01* and *Hsp68*), the expression levels and expression patterns of a large number of cytochrome P450 genes and some antioxidation-related genes were altered by an increase in temperature. These genes also play an important role in the high-temperature resistance of some insects [63,64,65], but whether they are meaningful in the high-temperature resistance process of *H. variegata* still needs to be confirmed.

## 5. Conclusions

We used high-throughput sequencing technology to compare the transcriptomes of *H. variegata* under different levels of high-temperature stress (32, 35, and 38 °C), which increased our understanding of the genome resources of *H. variegata* and provided a sound basis for further research. The number and function of DEGs in both temperature groups (32 vs. 35 °C and 35 vs. 38 °C) were different, indicating that H. variegate will mobilize different genes to cope with different high temperatures. The GO and KEGG enrichment analysis results showed that a large number of DEGs were involved in pathways, such as “Catalytic activity”, “Oxidoreductase activity”, “Metabolic pathways”, and “Longevity regulating pathway-multiple species”. Combined with the qRT-PCR results, the authenticity of transcriptome sequencing was confirmed. It has been demonstrated that genes involved in these pathways play an important role in the thermotolerance of *H. variegata*. Finally, we successfully interfered with the heat shock proteins (HSP70-01 and Hsp68) in *H. variegata* through the microinjection of dsRNA. Adult survival, longevity, and fecundity were lower in the group in which gene expression of the heat shock proteins (*Hsp70-01* and *Hsp68*) had been suppressed than in the control group (injection ds-*GFP*) at all experimental temperatures (32, 35, and 38 °C), which proved the key role of the heat shock proteins (*Hsp70-01* and *Hsp68*) in resistance to high-temperature stress in *H. variegata*. This study fills the gap in the research on the molecular mechanism on heat resistance in *H. variegate*.

## Figures and Tables

**Table 1:** Primers used in qRT-PCR.

Gene	Right Primer (5′–3′)	Reverse Primer (5′–3′)	E	*R* ^2^	Purpose
*P450-01*	AGGCATTCACCACGATCCAG	AAACTCTTGGCCCTTCTCCG	1.9648	99.97%	qRT-PCR
*P450-02*	TGGACTGCTCCAGAGGATTG	TCATGAGGCAGCTCTTTCCA	1.9687	99.45%	
*P450-03*	CAAAGTTGGATTGGCAGCCC	TTTTTCTGTCGCAATGCTGC	1.9435	99.64%	
*P450-04*	TGCTGAAGGAAGTTGATGCTCT	ACGCCTGTCATTTCTGCAGA	1.9760	99.91%	
*P450-05*	GATGCCGACGCCTATTAGCT	AGAGACCCAAACGCCGAATT	2.0024	99.77%	
*P450-06*	TCTGAAGCTTGATGAGTCACCA	TGGCTTTTGTGGCACTCGTA	1.9430	99.73%	
*Hsp70-01*	AACCCTGACGAAGCAGTAGC	TTGGTCATGACTCCACCTGC	1.9592	99.97%	
*Hsp70-02*	CAACCTTGAAGGGCCAATGC	AGCGATGAATCCCAGCAACA	1.9778	99.91%	
*Hsp68*	TGGTGGCTGAAGCAGAGAAG	GCTCAACTTGCTGCCACAAT	1.9743	99.73%	
*EF1α*	AGCCAACATTACCACTGA	GTATCCACGACGCAATTC	1.9966	99.43%	
*ds-Hsp70-01*	TGGCACAGTGATGACAGCAT	ACCCCAAGTTATGTGGCGTT	--	--	RNAi
*ds-Hsp68*	ATGCGAAACGTCTCATCGGAA	GTTCTTAGTCGACGCAGGCT	--	--	
*ds-GFP*	TGGTCCCAATTCTCGTGGAAC	CTTGAAGTTGACCTTGATGCC	--	--	

E stands for amplification efficiency; *R*^2^ stands for correlation coefficient.

**Table 2:** Quality control for sequence data from *Hippodamia variegata* under different levels of temperature stress.

ID	Raw Reads	Clean Reads	Error Rate	Q20	Q30	GC Content
32 °C-1	25,908,610	24,488,975	0.03%	97.93%	94.03%	41.06%
32 °C-2	28,820,184	27,389,182	0.03%	97.89%	93.98%	41.21%
32 °C-3	29,288,207	27,600,521	0.03%	97.85%	93.88%	40.61%
35 °C-1	28,403,718	26,967,327	0.02%	98.06%	94.35%	41.22%
35 °C-2	27,939,525	26,536,254	0.02%	98.09%	94.39%	41.30%
35 °C-3	29,349,401	27,911,084	0.02%	98.08%	94.36%	41.26%
38 °C-1	21,953,796	20,974,286	0.03%	97.91%	94.02%	41.11%
38 °C-2	28,418,287	27,094,748	0.02%	98.03%	94.28%	41.44%
38 °C-3	28,719,964	27,425,348	0.02%	98.05%	94.29%	40.70%
32 °C-1	25,908,610	24,488,975	0.03%	97.93%	94.03%	41.06%
32 °C-2	28,820,184	27,389,182	0.03%	97.89%	93.98%	41.21%

**Table 3:** Number of all annotated unigenes of *Hippodamia variegata* in seven databases.

Database	Number of Unigenes	Percentage (%)
Annotated in NR	23,616	34.45
Annotated in NT	7212	10.52
Annotated in KO	2747	4.01
Annotated in SwissProt	15,761	22.99
Annotated in Pfam	21,268	31.03
Annotated in GO	11,604	16.93
Annotated in COG/KOG	14,801	21.59
Annotated in all databases	762	1.11
Annotated in at least one database	28,651	41.80
Total unigenes	68,546	100

**Table 4:** The degree of gene expression at several time points after the injection of *Hipoddamia variagata* beetles with RNAi sequences designed to silence the Hsp70-01 and Hsp68 genes.

Tem	Group	ds-*Hsp70-01*			ds-*Hsp68*		
		24 h	48 h	72 h	24 h	48 h	72 h
32 °C	Control	1.01 ± 0.05	0.99 ± 0.05	0.99 ± 0.03	1.01 ± 0.05	0.99 ± 0.05	0.99 ± 0.03
	ds-target genes	0.62 ± 0.06 **	0.54 ± 0.03 **	0.65 ± 0.03 **	0.67 ± 0.06 **	0.63 ± 0.06 **	0.67 ± 0.08 *
35 °C	Control	1.01 ± 0.07	1.00 ± 0.08	1.00 ± 0.01	1.01 ± 0.07	1.00 ± 0.08	1.00 ± 0.01
	ds-target genes	0.51 ± 0.05 **	0.51 ± 0.04 **	0.65 ± 0.02 **	0.53 ± 0.05 **	0.51 ± 0.02 **	0.54 ± 0.04 **
38 °C	Control	1.04 ± 0.04	1.01 ± 0.05	1.02 ± 0.04	1.04 ± 0.04	1.01 ± 0.05	1.02 ± 0.04
	ds-target genes	0.51 ± 0.03 **	0.42 ± 0.01 **	0.52 ± 0.03 **	0.58 ± 0.01 **	0.56 ± 0.01 **	0.60 ± 0.01 **

Tem: Temperature. The results are shown as the means ± SE. The control was injected with the ds-*GFP*. The asterisk (*) behind each group indicates significant differences (Student’s *t*-test, *p* < 0.05) between the dsRNA treatments and the control. *, *p* < 0.05; **, *p* < 0.01.

## Data Availability

All data analyzed in this study are included in this article.

## Supplementary Materials

The following supporting information can be downloaded at https://www.mdpi.com/article/10.3390/insects15090678/s1: Figure S1. Pearson correlation between samples; Figure S2. The sequence information of *Hsp70-01*; Figure S3. The sequence information of *Hsp68*; Table S1. GO enrichment analysis (DEG number per pathway) of adults of *H. variegata* under different temperature stresses; Table S2. KEGG enrichment analysis (DEG number per pathway) of adults of *H. variegata* under different temperature stresses; Table S3. Statistics of FPKM values of DEGs at different temperatures of *Hippodamia variegata*.

## References

[1] Pachauri R.K., Meyer L.A., IPCC, Core Writing Team (2014). Climate Change 2014: Synthesis Report.

[2] Angilletta M.J., Huey R.B., Frazier M.R. (2010). Thermodynamic effects on organismal performance: Is hotter better?. Physiol. Biochem. Zool..

[3] Garrad R., Booth D.T., Furlong M.J. (2016). The effect of rearing temperature on development, body size, energetics and fecundity of the diamondback moth. Bull. Entomol. Res..

[4] Ardakani H.R., Samih M.A., Ravan S., Mokhtari A. (2020). Effect of temperature on the development and predatory potential of *Exochomus nigripennis* (Erichson) (Col.: Coccinellidae) fed on *Gossyparia spuria* (Modeer) (Hem.: Eriococcidae). Int. J. Trop. Insects.

[5] Mathew A., Morimoto R.I. (1998). Role of the heat shock response in the life and death of proteins. Ann. N. Y. Acad. Sci..

[6] Lopez-Martinez G., Elnitsky M.A., Benoit J.B., Lee R.E., Denlinger D.L. (2008). High resistance to oxidative damage in the Antarctic midge *Belgica antarctica*, and developmentally linked expression of genes encoding superoxide dismutase, catalase and heat shock proteins. Insect Biochem. Molec. Biol..

[7] Du R., Ma C.S., Zhao Q.H., Ma G., Yang H.P. (2007). Effects of heat stress on physiological and biochemical mechanisms of insects: A literature review. Acta Ecol. Sin..

[8] Ma G., Ma C.S. (2016). The impacts of extreme high temperature on insect populations under climate change: A review. Sci. Sin. Vitae.

[9] Ma C.S., Ma G., Pincebourde S. (2020). Survive a warming climate: Insect responses to extreme high temperatures. Annu. Rev. Entomol..

[10] Oliveira C.M.D., Silvatorres C.S.A.D., Torres J.B., Silva G.D.S. (2021). Estimation of population growth for two species of lady beetles (Coleoptera: Coccinellidae) under different temperatures. Biocontrol Sci. Technol..

[11] Kawakami Y., Yamazaki K., Ohashi K. (2014). Northward expansion and climatic factors affecting the distribution limits of *Cheilomenes sexmaculata* (Coleoptera: Coccinellidae) in Japan. Appl. Entomol. Zool..

[12] Yue J., He J., Zhang R., He D.H. (2009). Life tables of laboratory population of *Hippodamia variegata* at different temperatures. Chin. J. Appl. Entomol..

[13] Brown P.M.J., Roy H.E. (2015). Reflections on the long-term assessment of ladybird (Coleoptera: Coccinellidae) populations in the Czech Republic and the United Kingdom. Acta Soc. Zool. Bohemicae.

[14] Li X.L., Luo Y.L., Li H., Xie X., Ma R.H., Liu Y.J., Wang P.L., Lu Y.H. (2019). Regulation and control effects of Suaeda strips on the population occurrence of *Hippomidia variegata* in cotton fields. Xinjiang Agric. Sci..

[15] Sarkar N., Barik A. (2017). Effect of temperature on development and reproduction of *Epilachna dodecastigma* (Wied.) (Coleoptera: Coccinellidae). Proceed. Zool. Soc..

[16] Islam Y., Güncan A., Zhou X.M., Naeem A., Shah F.M. (2022). Effect of temperature on the life cycle of *Harmonia axyridis* (Pallas), and its predation rate on the *Spodoptera litura* (Fabricius) eggs. Sci. Rep..

[17] Kong X.X., Meng B.B., Guo P.F., Wang P.L. (2018). Predation functional response of adult of *Adonia variegata* (Goeze) to *Aphis gossypii* Glover at different temperatures. Xinjiang Agric. Sci..

[18] Arzigul R., Ding X.H., Tursun A., Yu G.Y., Fu K.Y., He J., Adili S., Guo W.C. (2021). Investigation and diversity of ladybug resources of farmland system in Xinjiang. J. Environ. Entomol..

[19] Yang Q., Liu J.P., Wyckhuys K.A.G., Yang Y.Z., Lu Y.H. (2022). Impact of heat stress on the predatory ladybugs *Hippodamia variegata* and *Propylaea quatuordecimpunctata*. Insects.

[20] Neven L.G. (2000). Physiological responses of insects to heat. Postharvest Biol. Technol..

[21] Zhang Y.H., Cai T.W., Ren Z.J., Liu Y., Yuan M.J., Cai Y.F., Yu C., Shu R.H., He S., Li J.H. (2021). Decline in symbiont-dependent host detoxification metabolism contributes to increased insecticide susceptibility of insects under high temperature. ISMEJ.

[22] Damos P., Savopoulou-Soultani M. (2011). Temperature-driven models for insect development and vital thermal requirements. Psyche J. Entomol..

[23] Liu Y.C., Su H., Li R.Q., Li X.T., Xu Y.S., Dai X.P., Zhou Y.Y., Wang H.B. (2017). Comparative transcriptome analysis of *Glyphodes pyloalis* Walker (Lepidoptera: Pyralidae) reveals novel insights into heat stress tolerance in insects. BMC Genom..

[24] Jiang F.Z., Zheng L.Y., Guo J.X., Zhang G.R. (2015). Effects of temperature stress on insect fertility and its physiological and biochemical mechanisms. J. Environ. Entomol..

[25] Mutz K.O., Heilkenbrinker A., Lönne M., Walter J.G., Stahl F. (2013). Transcriptome analysis using next-generation sequencing. Curr. Opin. Biotechnol..

[26] Zhang Q.L., Yuan M.L. (2013). Progress in insect transcriptomics based on the next-generation sequencing technique. Acta Entomol. Sin..

[27] Shen H.Y., He H., Lu C.D., Liang Y., Wu H.M., Zheng L.Z., Wang X.Y., Liang G.H. (2022). Comparative transcriptome analysis of two populations of *Dastarcus helophoroides* (Fairmaire) under high temperature stress. Forests.

[28] Ma W.H., Li X.Y., Shen J.S., Du Y.L., Xu K., Jiang Y.S. (2019). Transcriptomic analysis reveals *Apis mellifera* adaptations to high temperature and high humidity. Ecotox. Environ. Safe..

[29] Guo H.Z., Huang C.L., Jiang L., Cheng T.C., Feng T.S., Xia Q.Y. (2018). Transcriptome analysis of the response of silkworm to drastic changes in ambient temperature. Appl. Microbiol. Biotechnol..

[30] Ashraf H.J., Aguila L.C.R., Ahmed S., Haq I.U., Ali H., Ilyas M., Gu S.Y., Wang L.D. (2022). Comparative transcriptome analysis of *Tamarixia radiata* (Hymenoptera: Eulophidae) reveals differentially expressed genes upon heat shock. Comp. Biochem. Physiol. Part D Genom. Proteom..

[31] Yang Q., Liu J.P., Yang Y.Z., Lu Y.H. (2022). Transcriptome analysis of *Propylaea quatuordecimpunctata* L. (Coleoptera: Coccinellidae) under high temperature stress. Agriculture.

[32] Ren S.X., Wang X.M., Pang H., Peng Z.Q., Zeng T. (2009). Colored Pictorial Handbook of Ladybird Beetles in China.

[33] Gambino G., Perrone I., Gribaudo I. (2008). A rapid and effective method for RNA extraction from different tissues of grapevine and other woody plants. Phytochem. Anal..

[34] Grabherr M.G., Haas B.J., Yassour M., Levin J.Z., Thompson D.A., Amit I., Adiconis X., Fan L., Raychowdhury R., Zeng Q.D. (2011). Full-length transcriptome assembly from RNA-Seq data without a reference genome. Nat. Biotechnol..

[35] Pertea G., Huang X.Q., Liang F., Antonescu V., Sultana R., Karamycheva S., Lee Y., White J., Cheung F., Parvizi B. (2003). TIGR Gene indices clustering tools (TGICL): A software system for fast clustering of large EST datasets. Bioinformatics.

[36] Anders S., Huber W. (2010). Differential expression analysis for sequence count data. Genome Biol..

[37] Young M.D., Wakefield M.J., Smyth G.K., Oshlack A. (2010). Gene ontology analysis for RNA-seq: Accounting for selection bias. Genome Biol..

[38] Kanehisa M., Araki M., Goto S., Hattori M., Hirakawa M., Itoh M., Katayama T., Kawashima S., Okuda S., Tokimatsu T. (2008). KEGG for linking genomes to life and the environment. Nucleic Acids Res..

[39] Xie J.X., Liu T.H., Khashaveh A., Yi C.Q., Liu X.X., Zhang Y.J. (2021). Identification and evaluation of suitable reference genes for RT-qPCR analysis in *Hippodamia variegata* (Coleoptera: Coccinellidae) under different biotic and abiotic conditions. Front. Physiol..

[40] Livak K.J., Schmittgen T.D. (2001). Analysis of relative gene expression data using real-time quantitative PCR and the 2^−ΔΔCT^ method. Methods.

[41] Zhang S., Fu W.Y., Li N., Zhang F., Liu T.X. (2015). Antioxidant responses of *Propylaea japonica* (Coleoptera: Coccinellidae) exposed to high temperature stress. J. Insect Physiol..

[42] Michel D., Fiaboe K.K.M., Kekeunou S., Nanga S., Kuate A.F., Tonnang H.E.Z., Gnanvossou D., Hanna R. (2021). Temperature-based phenology model to predict the development, survival, and reproduction of the oriental fruit fly *Bactrocera dorsalis*. J. Therm. Biol..

[43] Liu Y.H., Li X.H., Yan X.F., Li G., Luo C.Y., He Y. (2022). Effects of short-term high temperatures on survival and reproduction of *Trabala vishnou gigantina* Yang (Lepidoptera: Lasiocampidae). Pak. J. Zool..

[44] Wang Z., Gerstein M., Snyder M. (2009). RNA-Seq: A revolutionary tool for transcriptomics. Nat. Rev. Genet..

[45] Quan N., Palfreyman R.W., Chan L.C.L., Reid S., Nielsen L.K. (2012). Transcriptome sequencing of and microarray development for a *Helicoverpa zea* cell line to investigate in vitro insect cell–baculovirus interactions. PLoS ONE.

[46] Lv J.H., Huang Z.E., Shi Y., Kang Y.L. (2020). Influences of different temperatures on the growth and reproduction of *Tribolium castaneum*. J. Chin. Cereals Oils Assoc..

[47] King A.M., Macrae T.H. (2015). Insect heat shock proteins during stress and diapause. Annu. Rev. Entomol..

[48] Lu M.X., Hua J., Cui Y.D., Du Y.Z. (2014). Five small heat shock protein genes from *Chilo suppressalis*: Characteristics of gene, genomic organization, structural analysis, and transcription profiles. Cell Stress Chaperones.

[49] Lu M.X., Li H.B., Zheng Y.T., Shi L., Du Y.Z. (2016). Identification, genomic organization and expression profiles of four heat shock protein genes in the western flower thrips, *Frankliniella occidentalis*. J. Therm. Biol..

[50] Zhang B., Leonard S.P., Li Y.Y., Moran N.A. (2019). Obligate bacterial endosymbionts limit thermal tolerance of insect host species. Proc. Natl. Acad. Sci. USA.

[51] Tang X.T., Sun M., Lu M.X., Du Y.Z. (2015). Expression patterns of five heat shock proteins in *Sesamia inferens* (Lepidoptera: Noctuidae) during heat stress. J. Asia-Pac. Entomol..

[52] Chen N., Tan J.Y., Wang Y., Qi M.H., Peng J.N., Chen D.X., Liu S., Li M.Y. (2022). A heat shock protein 70 protects the green peach aphid (*Myzus persicae*) against high-temperature stress. J. Asia-Pac. Entomol..

[53] Feder M.E., Hofmann G.E. (1999). Heat-shock proteins, molecular chaperones, and the stress response: Evolutionary and ecological physiology. Annu. Rev. Physiol..

[54] Lee G.J., Vierling E. (2000). A small heat shock protein cooperates with heat shock protein 70 systems to reactivate a heat-denatured protein1. Plant Physiol..

[55] Clare D.K., Saibil H.R. (2013). ATP-Driven molecular chaperone machines. Biopolymers.

[56] Boutet I., Tanguy A., Rousseau S., Auffret M., Moraga D. (2003). Molecular identification and expression of heat shock cognate 70 (*hsc70*) and heat shock protein 70 (*hsp70*) genes in the Pacific oyster *Crassostrea gigas*. Cell Stress Chaperones.

[57] Deane E.E., Woo N.Y.S. (2005). Cloning and characterization of the *hsp70* multigene family from silver sea bream: Modulated gene expression between warm and cold temperature acclimation. Biochem. Biophys. Res. Commun..

[58] Garbuz D.G., Yushenova I.A., Zatsepina O.G., Przhiboro A.A., Bettencourt B.R., Evgen’ev M.B. (2011). Organization and evolution of *hsp70* clusters strikingly differ in two species of Stratiomyidae (Diptera) inhabiting thermally contrasting environments. BMC Evol. Biol..

[59] Kourtidis A., Drosopoulou E., Nikolaidis N., Hatzi V.I., Chintiroglou C.C., Scouras Z.G. (2006). Identifcation of several cytoplasmic HSP70 genes from the Mediterranean mussel (*Mytilus galloprovincialis*) and their long-term evolution in Mollusca and Metazoa. J. Mol. Evol..

[60] Yu E.M., Yoshinaga T., Jalufka F.L., Ehsan H., Kaneko G. (2021). The complex evolution of the metazoan HSP70 gene family. Sci. Rep..

[61] Jin J.S., Zhao M., Wang Y., Zhou Z.S., Wan F.H., Guo J.Y. (2020). Induced thermotolerance and expression of three key Hsp genes (*Hsp70*, *Hsp21*, and *sHsp21*) and their roles in the high temperature tolerance of *Agasicles hygrophila*. Front. Physiol..

[62] Huang L.H., Bing C., Kang L. (2007). Impact of mild temperature hardening on thermotolerance, fecundity, and Hsp gene expression in *Liriomyza huidobrensis*. J. Insect Physiol..

[63] Yang H., Wang X.Y., Pei H.Y., Fan D. (2019). Cloning a peroxidase cDNA sequence from the Oriental Armyworm, *Mythimna separata* Walker and its induction to different temperature Stress. Chin. J. Biol. Control..

[64] Durak R., Dampc J., Kula-Maximenko M., Mołoń M., Durak T. (2021). Changes in antioxidative, oxidoreductive and detoxification enzymes during development of aphids and temperature increase. Antioxidants.

[65] Shen X.N., Liu W.X., Wan F.H., Lv Z.C., Guo J.Y. (2021). The role of cytochrome P450 4C1 and carbonic anhydrase 3 in response to temperature stress in *Bemisia tabaci*. Insects.

