# Supplementary material for: Heat Shock Protein 70 Genes Are Involved in the Thermal Tolerance of Hippodamia variegata
Source: Insects. 2024 Sep 8;15(9):678. doi: 10.3390/insects15090678 (PMC11431981; doi:10.3390/insects15090678)
Supplement: Supplementary file 1 [file insects-15-00678-s001.zip › insects-3147808-supplementary.pdf]

Supplemental Table S1. GO enrichment analysis (the DEGs number in per pathway) of adults of *H. variegata* under different temperature stress.

Groups	Pathway ID	DEGs		
		All	Up	Down
35 vs 32℃	metabolic process	304	92	212
	oxidation-reduction process	84	12	72
	proteolysis	46	18	28
	DNA integration	23	10	13
	DNA alkylation	3	2	1
	DNA methylation	3	2	1
	DNA methylation or demethylation	3	2	1
	outer membrane	12	3	9
	catalytic activity	296	81	215
	oxidoreductase activity	85	12	73
	cation binding	85	35	50
	metal ion binding	84	35	49
	cofactor binding	71	11	60
	transition metal ion binding	61	25	36
	coenzyme binding	48	7	41
	peptidase activity, acting on L-amino acid peptides	45	16	29
	peptidase activity	45	16	29
	oxidoreductase activity, acting on CH-OH group of donors	30	1	29
	iron ion binding	21	6	15
	heme binding	20	2	18
	tetrapyrrole binding	20	2	18
	flavin adenine dinucleotide binding	18	1	17
	oxidoreductase activity, acting on paired donors, with incorporation or reduction of molec…	18	3	15
	oxidoreductase activity, acting on the CH-OH group of donors, NAD or NADP as acceptor	13	1	12
	pyridoxal phosphate binding	11	4	7
	vitamin B6 binding	11	4	7
	vitamin binding	11	4	7
	carboxy-lyase activity	9	3	6
	oxidoreductase activity, acting on diphenols and related substances as donors, oxygen as a…	4	-	4
	mannose-6-phosphate isomerase activity	2	-	2
38 vs 35℃	DNA integration	20	15	5
	drug metabolic process	15	11	4
	lipid transport	13	12	1
	lipid localization	13	12	1
	steroid metabolic process	7	3	4
	small molecule catabolic process	6	6	-
	alpha-amino acid metabolic process	6	6	-

	sterol metabolic process	5	2	3
	cellular amino acid catabolic process	5	5	-
	organic acid catabolic process	5	5	-
	carboxylic acid catabolic process	5	5	-
	aromatic amino acid family catabolic process	4	4	-
	sterol biosynthetic process	4	1	3
	tryptophan catabolic process	2	2	-
	amine catabolic process	2	2	-
	tryptophan catabolic process to kynurenine	2	2	-
	catalytic activity	216	132	84
	oxidoreductase activity	53	42	11
	oxidoreductase activity, acting on paired donors, with incorporation or reduction of molec…	19	18	1
	heme binding	18	17	1
	tetrapyrrole binding	18	17	1
	iron ion binding	16	15	1
	lipid transporter activity	10	10	-
	oxidoreductase activity, acting on single donors with incorporation of molecular oxygen, i…	5	5	-
	phosphoribosylaminoimidazolecarboxamide formyltransferase activity	3	3	-
	IMP cyclohydrolase activity	3	3	-
	cyclohydrolase activity	3	3	-
	hydroxymethyl-, formyl- and related transferase activity	3	3	-
	oxidoreductase activity, acting on paired donors, with incorporation or reduction of molecu…	3	3	-
	tryptophan 2,3-dioxygenase activity	2	2	-
38 vs 32℃	protein metabolic process	97	26	71
	Proteolysis	57	10	47
	cell projection organization	8	2	6
	cell projection assembly	5	1	4
	cell morphogenesis	5	2	3
	cellular component morphogenesis	5	2	3
	tissue development	5	2	3
	pilus assembly	4	1	3
	pilus organization	4	1	3
	pyrimidine nucleoside metabolic process	3	1	2
	proteasome-mediated ubiquitin-dependent protein catabolic process	3	2	1
	outer membrane	11	6	5
	catalytic activity	250	66	184
	hydrolase activity	121	28	93
	catalytic activity, acting on a protein	77	19	58
	peptidase activity	59	9	50

peptidase activity, acting on L-amino acid peptides	56	9	47
endopeptidase activity	36	9	27
serine-type peptidase activity	21	2	19
hydrolase activity, acting on acid phosphorus-nitrogen bonds	21	2	19
serine hydrolase activity	21	2	19
exopeptidase activity	12	-	12
aspartic-type endopeptidase activity	11	6	5
aspartic-type peptidase activity	11	6	5
carboxypeptidase activity	9	-	9
odorant binding	8	2	6
serine-type exopeptidase activity	7	-	7
serine-type carboxypeptidase activity	6	-	6
mannose-6-phosphate isomerase activity	2	1	1
palmitoyl hydrolase activity	2	-	2

Note: "All" indicates the number of all DEGs; "Up" indicates the number of up-regulated genes; "Down" indicates the number of down-regulated genes; "- "indicates no DEGs.

Supplemental Table S2. KEGG enrichment analysis (the DEGs number in per pathway) of adults of *H. variegata* under different temperature stress.

Groups	Pathway ID	DEGs		
		All	Up	Down
35 vs 32℃	Valine, leucine and isoleucine degradation	8	-	8
	Tryptophan metabolism	7	1	6
	Steroid hormone biosynthesis	9	-	9
	Retinol metabolism	12	1	11
	Metabolic pathways	102	15	87
	Lysosome	13	1	12
	Histidine metabolism	7	1	6
	Glycine, serine and threonine metabolism	29	-	29
	Glycerolipid metabolism	12	1	11
	Fatty acid metabolism	9	1	8
	Fatty acid degradation	7	-	7
	Fat digestion and absorption	10	-	10
	Drug metabolism - other enzymes	11	3	8
	Chemical carcinogenesis	13	-	13
	Biosynthesis of amino acids	12	1	11
	beta-Alanine metabolism	11	2	9
	Ascorbate and aldarate metabolism	8	1	7
	Arginine and proline metabolism	9	-	9
	Amino sugar and nucleotide sugar metabolism	11	2	9
	ABC transporters	8	-	8
38 vs 35℃	Vitamin B6 metabolism	4	4	-
	Valine, leucine and isoleucine degradation	8	8	-
	Tryptophan metabolism	11	11	-
	Pyruvate metabolism	7	6	1
	Phenylalanine, tyrosine and tryptophan biosynthesis	4	4	-
	Phenylalanine metabolism	6	6	-
	One carbon pool by folate	7	7	-
	Metabolic pathways	90	75	15
	Longevity regulating pathway - multiple species	11	-	11
	Legionellosis	9	-	9
	Glyoxylate and dicarboxylate metabolism	6	6	-
	Glycine, serine and threonine metabolism	15	14	1
	Fatty acid metabolism	8	5	3
	Fatty acid degradation	8	7	1
	Estrogen signaling pathway	12	2	10
	Drug metabolism - other enzymes	8	6	2
	Carbon metabolism	14	14	-
	Biosynthesis of amino acids	15	15	-
	Antigen processing and presentation	11	-	11
	ABC transporters	8	3	5

38 vs 32℃	Vitamin digestion and absorption	5	2	3
	Vitamin B6 metabolism	4	4	-
	Spliceosome	14	1	13
	Renin secretion	8	3	5
	Protein processing in endoplasmic reticulum	15	3	12
	Pentose and glucuronate interconversions	5	-	5
	Metabolic pathways	73	16	57
	Lysosome	10	-	9
	Longevity regulating pathway - multiple species	11	1	10
	Legionellosis	8	-	8
	Glycine, serine and threonine metabolism	15	5	10
	Fatty acid metabolism	12	2	10
	Fatty acid degradation	6	1	5
	Fat digestion and absorption	7	1	6
	Estrogen signaling pathway	13	5	8
	ECM-receptor interaction	10	4	6
	Biosynthesis of amino acids	10	6	4
	Bile secretion	7	2	5
	Antigen processing and presentation	11	-	11
	ABC transporters	8	1	7

Note: “All” indicates the number of all DEGs; “Up” indicates the number of up-regulated genes; “Down” indicates the number of down-regulated genes; “- “indicates no DEGs

Supplemental Tables S3. Statistics of FPKM values of DEGs at different temperatures of *Hippodamia variegata*.

Gene ID	FPKM Value at three temperatures			$F_{2,6}$	P
	32℃	35℃	38℃		
cytochrome P450 (*P450-01*)	4.51±0.06	14.24±0.22	4.85±0.07	3131.61	<0.001
cytochrome P450 (*P450-02*)	2.31±0.12	5.51±0.12	2.32±0.19	305.91	<0.001
cytochrome P450 (*P450-03*)	1.44±0.07	3.55±0.14	3.17±0.18	140.29	<0.001
cytochrome P450 (*P450-04*)	1.06±0.06	4.26±0.15	1.17±0.06	643.83	<0.001
cytochrome P450 (*P450-05*)	1.85±0.10	6.74±0.17	6.95±0.10	999.15	<0.001
cytochrome P450 (*P450-06*)	0.89±0.03	2.23±0.10	0.93±0.04	277.32	<0.001
Heat shock protein 70 (*Hsp70-01*)	6.32±0.11	5.84±0.11	20.44±0.37	2546.88	<0.001
Heat shock protein 70 (*Hsp70-02*)	7.37±0.20	7.98±0.07	32.10±0.65	2536.11	<0.001
Heat shock protein 70 (*Hsp68*)	3.28±0.19	19.93±0.11	19.12±0.11	8388.85	<0.001
cytochrome P450 (*P450-01*)	4.51±0.06	14.24±0.22	4.85±0.07	3131.61	<0.001
cytochrome P450 (*P450-02*)	2.31±0.12	5.51±0.12	2.32±0.19	305.91	<0.001

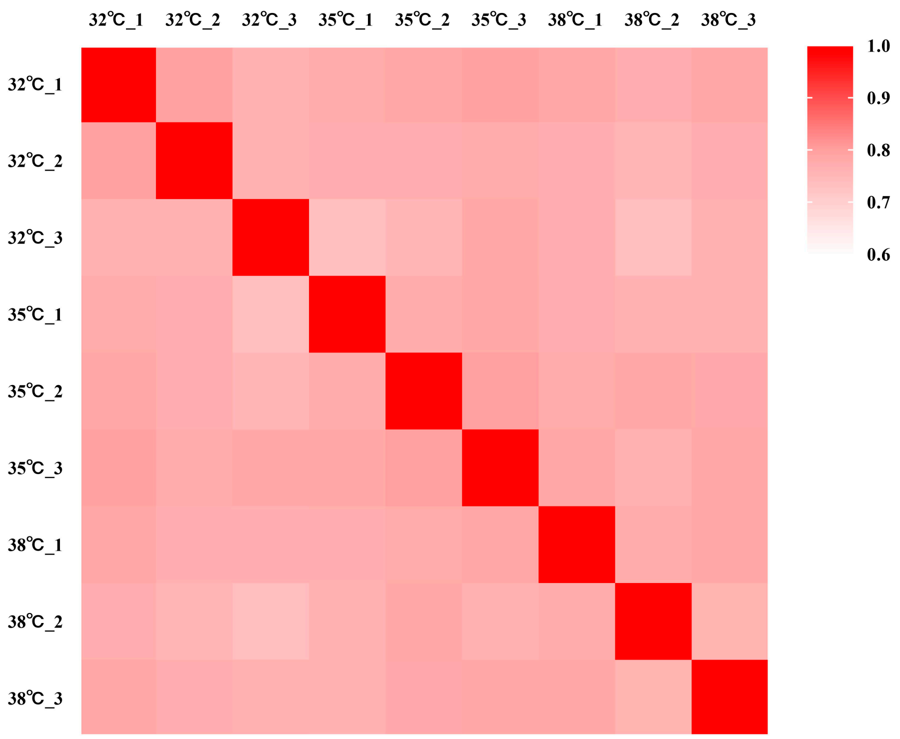

Supplemental Figure S1. Pearson correlation between samples.

```
                10        20        30        40        50        60        70        80        90
1       ATGGTTAAAGCTCCAGCAATTGGTATCGACTTGGGAACTACATATTCCTGCGTCGGTGTATGGCAACATGGAAAAGTAGAGATCATCGCA
1        M  V  K  A  P  A  I  G  I  D  L  G  T  T  Y  S  C  V  G  V  W  Q  H  G  K  V  E  I  I  A

               100       110       120       130       140       150       160       170       180
91      AACGATCAAGGAAACAGGACAACACCAAGTTATGTGGCTTTCACAGACACGGAGAGACTCCTTGGAGATGCCGCGAAGAACCAGGTGGCG
31       N  D  Q  G  N  R  T  T  P  S  Y  V  A  F  T  D  T  E  R  L  L  G  D  A  A  K  N  Q  V  A

               190       200       210       220       230       240       250       260       270
181     ATGAACCCCAGCAACACAGTTTTCGATGCGAAACGTCTCATCGGAAGGAAATACGACGATCCAAAAATCCAGCAAGACATCAAACACTGG
61       M  N  P  S  N  T  V  F  D  A  K  R  L  I  G  R  K  Y  D  D  P  K  I  Q  Q  D  I  K  H  W

               280       290       300       310       320       330       340       350       360
271     CCGTTCAAAGTCATCAGCGAGAGCGGCAAGCCGAAAATCCAAGTAGAATTCAAGGGAGAAAAAAAGGTATTTGCACCAGAAGAAATCAGC
91       P  F  K  V  I  S  E  S  G  K  P  K  I  Q  V  E  F  K  G  E  K  K  V  F  A  P  E  E  I  S

               370       380       390       400       410       420       430       440       450
361     TCAATGGTACTCACAAAAATGAAAGAAACCGCAGAAGCATATTTAGGTACAACGGTTAAAGACGCAGTTATCACAGTTCCAGCATATTTC
121      S  M  V  L  T  K  M  K  E  T  A  E  A  Y  L  G  T  T  V  K  D  A  V  I  T  V  P  A  Y  F

               460       470       480       490       500       510       520       530       540
451     AACGACTCACAAAGACAGGCAACAAAAGACGCAGGTGTCATCGCTGGTTTGAATGTGATGAGGATAATAAATGAACCGACAGCAGCTGCG
151      N  D  S  Q  R  Q  A  T  K  D  A  G  V  I  A  G  L  N  V  M  R  I  I  N  E  P  T  A  A  A

               550       560       570       580       590       600       610       620       630
541     CTGGCCTATGGATTGGACAAAAACCTTAAAGGTGAAAGAAATGTTCTTATCTTTGACCTGGGTGGAGGAACATTCGATGTTTCTATACTG
181      L  A  Y  G  L  D  K  N  L  K  G  E  R  N  V  L  I  F  D  L  G  G  G  T  F  D  V  S  I  L

               640       650       660       670       680       690       700       710       720
631     ACAATCGATGAGGGAAGTCTGTTCGAAGTACGAGCCACTGCTGGTGATACACACCTTGGTGGTGAAGACTTCGACAATAGACTAGTCAAT
211      T  I  D  E  G  S  L  F  E  V  R  A  T  A  G  D  T  H  L  G  G  E  D  F  D  N  R  L  V  N

               730       740       750       760       770       780       790       800       810
721     CACCTGGCAGATGAATTCAAAAGGAAATTCCGAAAAGACCTACGGAACAACCCAAGAAGCCTGCGTCGACTAAGAACAGCAGCAGAACGA
241      H  L  A  D  E  F  K  R  K  F  R  K  D  L  R  N  N  P  R  S  L  R  R  L  R  T  A  A  E  R

               820       830       840       850       860       870       880       890       900
811     GCCAAACGTACACTGTCATCAAGTACGGAAGCAACGATTGAAATAGATGCATTGTTCGATGGAATCGACTTCTACACCAAAGTTACAAGA
271      A  K  R  T  L  S  S  S  T  E  A  T  I  E  I  D  A  L  F  D  G  I  D  F  Y  T  K  V  T  R

               910       920       930       940       950       960       970       980       990
901     GCAAGGTTCGAAGAACTTTGCTCCGACTTATTCAGAGGAACATTACAACCAGTCGAGAAAGCATTGACAGATGCTAAGATGGACAAGGCA
301      A  R  F  E  E  L  C  S  D  L  F  R  G  T  L  Q  P  V  E  K  A  L  T  D  A  K  M  D  K  A

              1000      1010      1020      1030      1040      1050      1060      1070      1080
991     TCTATCCATGATGTTGTATTAGTTGGAGGTTCAACAAGAATTCCTAAGATCCAACAGCTTCTGCAAAATTACTTCTGCGGCAAATCATTA
331      S  I  H  D  V  V  L  V  G  G  S  T  R  I  P  K  I  Q  Q  L  L  Q  N  Y  F  C  G  K  S  L

              1090      1100      1110      1120      1130      1140      1150      1160      1170
1081    AATCTTTCCATCAACCCTGACGAAGCAGTAGCATACGGCGCAGCAGTCCAGGCAGCAGTATTGAACGGCGAGCAAGATTCCAAGATTCAA
361      N  L  S  I  N  P  D  E  A  V  A  Y  G  A  A  V  Q  A  A  V  L  N  G  E  Q  D  S  K  I  Q

              1180      1190      1200      1210      1220      1230      1240      1250      1260
1171    GATGTACTTCTTGTTGACGTTACACCATTATCACTGGGCATTGAAACGGCAGGTGGAGTCATGACCAAAATCATCGAGAGAAATGCAAGA
391      D  V  L  L  V  D  V  T  P  L  S  L  G  I  E  T  A  G  G  V  M  T  K  I  I  E  R  N  A  R

              1270      1280      1290      1300      1310      1320      1330      1340      1350
1261    ATTCCTTGTAAACAAACTCAAACATTCACTACGTATGCTGATAATCAACCTGCAGTTACCATCCAAGTGTTTGAAGGCGAAAGAGTCATG
421      I  P  C  K  Q  T  Q  T  F  T  T  Y  A  D  N  Q  P  A  V  T  I  Q  V  F  E  G  E  R  V  M

              1360      1370      1380      1390      1400      1410      1420      1430      1440
1351    ACCAAAGATAACAACCTGCTAGGAACCTTCGATCTAACTGGGCTTCCACCAGCCCCACGAGGAGTTCCAAAGATTGAGGTAACATTCGAT
451      T  K  D  N  N  L  L  G  T  F  D  L  T  G  L  P  P  A  P  R  G  V  P  K  I  E  V  T  F  D

              1450      1460      1470      1480      1490      1500      1510      1520      1530
1441    CTGGATGCCAATGGAATCTTGAACGTATCAGCCAAAGATTCCAGTTCAGGTAACTCCAAGAACATCACAATCAAAAACGATAAAGGAAGG
481      L  D  A  N  G  I  L  N  V  S  A  K  D  S  S  S  G  N  S  K  N  I  T  I  K  N  D  K  G  R

              1540      1550      1560      1570      1580      1590      1600      1610      1620
1531    TTGTCCCAGAAAGACATCGATAAGATGGTGGCTGAAGCAGAGAAGTACAAAGAAGAAGATGAGAAACAAAAACTGAGGATCGATGCTCGA
511      L  S  Q  K  D  I  D  K  M  V  A  E  A  E  K  Y  K  E  E  D  E  K  Q  K  L  R  I  D  A  R

              1630      1640      1650      1660      1670      1680      1690      1700      1710
1621    AACAAACTTGAAGCCTATGTTTTCCAACTCAAACAAGCAGTTCAAGATTGTGGCAGCAAGTTGAGCGAAGAAGACAAGTCCACTGTGGAA
541      N  K  L  E  A  Y  V  F  Q  L  K  Q  A  V  Q  D  C  G  S  K  L  S  E  E  D  K  S  T  V  E

              1720      1730      1740      1750      1760      1770      1780      1790      1800
1711    AGAGAATGCCAAAATTGCCTGCAGTGGCTAGACTCCAATACCCTGGCTGAGAAAGACGAGTACGAGGACAAACAGAAACAACTAACATCT
571      R  E  C  Q  N  C  L  Q  W  L  D  S  N  T  L  A  E  K  D  E  Y  E  D  K  Q  K  Q  L  T  S

              1810      1820      1830      1840      1850      1860      1870      1880      1890
1801    ATATGCAGTCCTATCATGGCCAAGTTATATGGAGGAGCTCAAAATGCTAACAACTTCGGTGGAATGCCTGGCAGCTGCGGACAACAAGCA
601      I  C  S  P  I  M  A  K  L  Y  G  G  A  Q  N  A  N  N  F  G  G  M  P  G  S  C  G  Q  Q  A

              1900      1910      1920      1930      1940
1891    GGTGGTGGCTTCGGAGGCAGACAAGGAGGACCTACCATTGAAGAAGTCGATTAA
631      G  G  G  F  G  G  R  Q  G  G  P  T  I  E  E  V  D  *
```

Supplemental Figure S2. The sequence information of *Hsp70-01*.

```
          10        20        30        40        50        60        70        80        90
1     ATGGTTAAAGCTCCAGCAATTGGTATCGACTTGGGAACTACATATTCCTGCGTCGGTGTATGGCAACATGGAAAAGTAGAGATCATCGCA
1      M  V  K  A  P  A  I  G  I  D  L  G  T  T  Y  S  C  V  G  V  W  Q  H  G  K  V  E  I  I  A

         100       110       120       130       140       150       160       170       180
91    AACGATCAAGGAAACAGGACAACACCAAGTTATGTGGCTTTCACAGACACGGAGAGACTCCTTGGAGATGCCGCGAAGAACCAGGTGGCG
31     N  D  Q  G  N  R  T  T  P  S  Y  V  A  F  T  D  T  E  R  L  L  G  D  A  A  K  N  Q  V  A

         190       200       210       220       230       240       250       260       270
181   ATGAACCCCAGCAACACAGTTTTCGATGCGAAACGTCTCATCGGAAGGAAATACGACGATCCAAAAATCCAGCAAGACATCAAACACTGG
61     M  N  P  S  N  T  V  F  D  A  K  R  L  I  G  R  K  Y  D  D  P  K  I  Q  Q  D  I  K  H  W

         280       290       300       310       320       330       340       350       360
271   CCGTTCAAAGTCATCAGCGAGAGCGGCAAGCCGAAAATCCAAGTAGAATTCAAGGGAGAAAAAAAGGTATTTGCACCAGAAGAAATCAGC
91     P  F  K  V  I  S  E  S  G  K  P  K  I  Q  V  E  F  K  G  E  K  K  V  F  A  P  E  E  I  S

         370       380       390       400       410       420       430       440       450
361   TCAATGGTACTCACAAAAATGAAAGAAACCGCAGAAGCATATTTAGGTACAACGGTTAAAGACGCAGTTATCACAGTTCCAGCATATTTC
121    S  M  V  L  T  K  M  K  E  T  A  E  A  Y  L  G  T  T  V  K  D  A  V  I  T  V  P  A  Y  F

         460       470       480       490       500       510       520       530       540
451   AACGATTCACAAAGACAGGCAACAAAAGACGCAGGTGTCATCGCTGGTTTGAATGTGATGAGGATAATAAATGAACCGACAGCAGCTGCG
151    N  D  S  Q  R  Q  A  T  K  D  A  G  V  I  A  G  L  N  V  M  R  I  I  N  E  P  T  A  A  A

         550       560       570       580       590       600       610       620       630
541   CTGGCCTATGGATTGGACAAAAACCTTAAAGGTGAAAGAAATGTTCTTATCTTTGACCTGGGTGGAGGAACATTCGATGTTTCTATACTC
181    L  A  Y  G  L  D  K  N  L  K  G  E  R  N  V  L  I  F  D  L  G  G  G  T  F  D  V  S  I  L

         640       650       660       670       680       690       700       710       720
631   ACAATTGATGAGGGAAGTCTGTTCGAAGTACGAGCCACTGCTGGTGATACACACCTTGGTGGTGAAGACTTCGACAATAGACTAGTCAAT
211    T  I  D  E  G  S  L  F  E  V  R  A  T  A  G  D  T  H  L  G  G  E  D  F  D  N  R  L  V  N

         730       740       750       760       770       780       790       800       810
721   CACCTGGCAGATGAATTCAAAAGGAAATTCCGAAAAGACCTACGGAACAACCCAAGAAGCCTGCGTCGACTAAGAACAGCAGCAGAACGA
241    H  L  A  D  E  F  K  R  K  F  R  K  D  L  R  N  N  P  R  S  L  R  R  L  R  T  A  A  E  R

         820       830       840       850       860       870       880       890       900
811   GCCAAACGTACACTGTCATCAAGTACGGAAGCAACGATTGAAATAGATGCATTGTTCGATGGAATTGACTTCTACACCAAAGTTACAAGA
271    A  K  R  T  L  S  S  S  T  E  A  T  I  E  I  D  A  L  F  D  G  I  D  F  Y  T  K  V  T  R

         910       920       930       940       950       960       970       980       990
901   GCAAGGTTCGAGGAACTTTGCTCCGACTTATTCAGAGGAACATTACAACCAGTCGAGAAAGCATTGACAGATGCTAAGATGGACAAGGCA
301    A  R  F  E  E  L  C  S  D  L  F  R  G  T  L  Q  P  V  E  K  A  L  T  D  A  K  M  D  K  A

        1000      1010      1020      1030      1040      1050      1060      1070      1080
991   TCTATCCATGATGTTGTATTAGTTGGAGGTTCAACAAGAATTCCTAAGATCCAACAGCTTCTGCAAAATTACTTCTGCGGCAAATCATTA
331    S  I  H  D  V  V  L  V  G  G  S  T  R  I  P  K  I  Q  Q  L  L  Q  N  Y  F  C  G  K  S  L

        1090      1100      1110      1120      1130      1140      1150      1160      1170
1081  AATCTTTCCATCAACCCTGACGAAGCAGTAGCATACGGCGCAGCAGTCCAGGCAGCAGTATTGAACGGCGAGCAAGATTCCAAGATTCAA
361    N  L  S  I  N  P  D  E  A  V  A  Y  G  A  A  V  Q  A  A  V  L  N  G  E  Q  D  S  K  I  Q

        1180      1190      1200      1210      1220      1230      1240      1250      1260
1171  GACGTACTTCTTGTTGACGTTACACCATTATCATTGGGCATTGAAACGGCAGGTGGAGTCATGACCAAAATCATCGAGAGAAATGCAAGA
391    D  V  L  L  V  D  V  T  P  L  S  L  G  I  E  T  A  G  G  V  M  T  K  I  I  E  R  N  A  R

        1270      1280      1290      1300      1310      1320      1330      1340      1350
1261  ATTCCTTGTAAACAAACTCAAACATTCACTACGTATGCTGATAATCAACCTGCAGTTACCATCCAAGTGTTTGAAGGCGAAAGAGTCATG
421    I  P  C  K  Q  T  Q  T  F  T  T  Y  A  D  N  Q  P  A  V  T  I  Q  V  F  E  G  E  R  V  M

        1360      1370      1380      1390      1400      1410      1420      1430      1440
1351  ACCAAAGATAACAACCTGCTAGGAACCTTTGATCTAACTGGGCTTCCACCAGCCCCACGAGGAGTGCCAAAGATTGAGGTAACATTCGAT
451    T  K  D  N  N  L  L  G  T  F  D  L  T  G  L  P  P  A  P  R  G  V  P  K  I  E  V  T  F  D

        1450      1460      1470      1480      1490      1500      1510      1520      1530
1441  CTGGATGCCAATGGAATCTTGAACGTATCAGCCAAAGATTCCAGTTCAGGTAACTCCAAGAACATCACAATCAAAAACGATAAAGGAAGG
481    L  D  A  N  G  I  L  N  V  S  A  K  D  S  S  S  G  N  S  K  N  I  T  I  K  N  D  K  G  R

        1540      1550      1560      1570      1580      1590      1600      1610      1620
1531  TTGTCCCAGAAAGACATCGATAAGATGGTGGCTGAAGCAGAGAAGTACAAAGAAGAAGATGAGAAACAAAAACTGAGGATCGATGCTCGA
511    L  S  Q  K  D  I  D  K  M  V  A  E  A  E  K  Y  K  E  E  D  E  K  Q  K  L  R  I  D  A  R

        1630      1640      1650      1660      1670      1680      1690      1700      1710
1621  AACAAACTTGAAGCCTATGTTTTCCAACTCAAACAAGCAGTTCAAGATTGTGGCAGCAAGTTGAGCGAAGAAGACAAGTCCCCTGTGGAA
541    N  K  L  E  A  Y  V  F  Q  L  K  Q  A  V  Q  D  C  G  S  K  L  S  E  E  D  K  S  P  V  E

        1720      1730      1740      1750      1760      1770      1780      1790      1800
1711  AGAGAATGCCAAAATTGCCTGCAGTGGCTAGACTCCAATACCCTGGCTGAGAAAGAAGAGTACGAGGACAAACAGAAACAACTAACATCT
571    R  E  C  Q  N  C  L  Q  W  L  D  S  N  T  L  A  E  K  E  E  Y  E  D  K  Q  K  Q  L  T  S

        1810      1820      1830      1840      1850      1860      1870      1880      1890
1801  ATATGCAGTCCTATCATGGCCAAGTTATATGGAGGAGCTCAAAATGCTAACAACTTCGGTGGAATGCCTGGCAGCTGCGGACAACAAGCA
601    I  C  S  P  I  M  A  K  L  Y  G  G  A  Q  N  A  N  N  F  G  G  M  P  G  S  C  G  Q  Q  A

        1900      1910      1920      1930      1940
1891  GGTGGTGGCTTCGGAGGCAGACAAGGAGGACCTACCATTGAAGAAGTCGATTAA
631    G  G  G  F  G  G  R  Q  G  G  P  T  I  E  E  V  D  *
```

Supplemental Figure S3. The sequence information of *Hsp68*.
